# Pulmonary MALT lymphoma diagnosed with transbronchial lung cryobiopsy after unsuccessful transbronchial lung biopsy: a case report

**DOI:** 10.3389/fmed.2025.1596730

**Published:** 2025-06-17

**Authors:** Akinari Tsukada, Manabu Suzuki, Kento Misumi, Hideki Miyazaki, Toru Igari, Jin Takasaki, Naoki Nishimura, Hiroshi Nokihara, Shinyu Izumi, Masayuki Hojo

**Affiliations:** ^1^Department of Respiratory Medicine, National Center for Global Health and Medicine, Tokyo, Japan; ^2^Department of Pathology, National Center for Global Health and Medicine, Tokyo, Japan

**Keywords:** MALT lymphoma, bronchoscopy, cryobiopsy, non-Hodgkin lymphoma, diagnostic yield

## Abstract

Primary pulmonary mucosa-associated lymphoid tissue (MALT) lymphoma is a rare primary lung neoplasm and a low-grade B-cell non-Hodgkin lymphoma subtype. Although the prognosis is generally favorable, nonsurgical biopsy methods often have a low diagnostic yield, leaving the optimal approach for pulmonary lesion biopsy unclear. This case study illustrates the successful diagnosis of pulmonary MALT lymphoma using transbronchial lung cryobiopsy (TBLC) after usual transbronchial lung biopsy (TBLB) failed to provide a definitive diagnosis. A 47-year-old female with no significant medical history was referred to our institution after a chest radiograph revealed abnormalities. Subsequent chest computed tomography showed tumorous lesions measuring 20 mm in the right middle lobe and 35 mm in the left lower lobe, prompting a bronchoscopic examination. Guided sheath transbronchial lung biopsy (GS-TBLB) was performed on these two lesions, revealing CD20-positive lymphocytic infiltration. However, the lack of a clear light chain restriction and minimal plasma cell differentiation did not suggest malignant lymphoma. Considering cryptogenic organizing pneumonia, prednisolone was administered for 3 months, but no changes in radiographic findings were observed. Therefore, a second bronchoscopic examination using TBLC was planned. TBLC was performed twice on the left lower lobe lesion, revealing diffuse infiltration of small lymphoid cells into the lung parenchyma and subepithelial bronchial tissue, with some cells differentiating into plasma cells. *In situ* hybridization showed *λ* dominant light chain restriction, confirming the diagnosis of MALT lymphoma. The patient, asymptomatic and with no evidence of tumor cell infiltration into the bone marrow, continued to be observed. Pulmonary MALT lymphoma has a favorable prognosis and less invasive diagnostic approaches are desirable. However, conventional TBLB has a low diagnostic yield, suggesting the potential utility of TBLC in diagnosis.

## Introduction

Mucosa-associated lymphoid tissue (MALT) lymphoma is classified within a group of marginal zone B-cell lymphomas, which includes nodal and splenic marginal zone B-cell lymphomas. MALT lymphomas typically occur in the stomach, salivary glands, lungs, and thyroid gland (1). Approximately 8% of non-Hodgkin’s lymphomas are MALT (2). MALT lymphoma arises from chronic antigen stimulation by self-antigens or microbial sources (3).

Although rare, pulmonary MALT lymphoma accounts for about 80% of all primary pulmonary lymphoma cases (4, 5). Nearly half of the patients with MALT lymphoma are asymptomatic at diagnosis, with abnormalities often detected via chest radiography or CT scans (6).

Conventional bronchoscopic and CT-guided biopsies have low diagnostic rates, often necessitating a surgical lung biopsy for a definitive diagnosis (7). Furthermore, surgical and computed tomography-guided biopsies are highly invasive. Therefore, obtaining larger tissue samples using minimally invasive diagnostic methods is important.

Transbronchial lung cryobiopsy (TBLC) is a relatively new technique that results in fewer crush artifacts and allows for the collection of larger lung tissue samples. It is increasingly utilized in diagnosing interstitial lung diseases. Several reports have demonstrated the effectiveness of cryobiopsy in diagnosing lung lymphoproliferative disorders (8–11). Here, we present a case of primary pulmonary MALT lymphoma where a definitive diagnosis was achieved through TBLC after a conventional transbronchial lung biopsy failed.

## Case presentation

**Figure 1:**
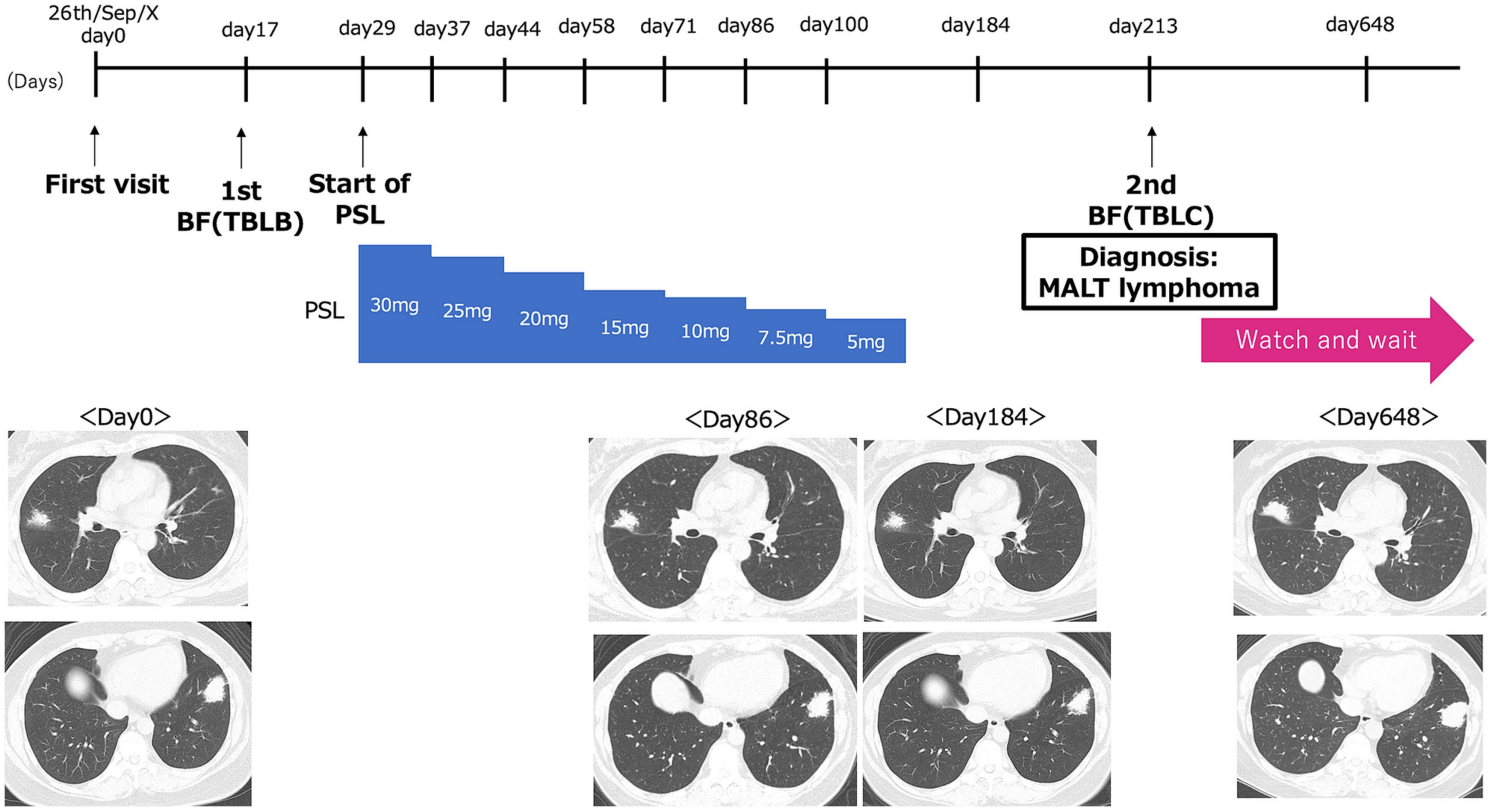
Graphical timeline of the present case. Day 0 corresponds to the patient’s initial visit to our hospital on September 26, Year X. The timeline shows the dates of the first bronchoscopy with transbronchial lung biopsy [1st BF (TBLB)], the initiation of prednisolone (Start of PSL), and the second bronchoscopy with transbronchial lung cryobiopsy [2nd BF (TBLC)]. Representative CT images at each of these time points are shown below.

The patient was a healthy 47-year-old woman who was referred to our hospital in September X-year after an abnormality was noted on chest radiography without any symptoms. The patient was a homemaker with no prior medical history or comorbidities, no smoking history, and no medication use. She also had no family history of lymphoma or other malignancies. Physical examination revealed no apparent abnormalities. The entire clinical course of this patient is shown in Figure 1.

**Figure 2:**
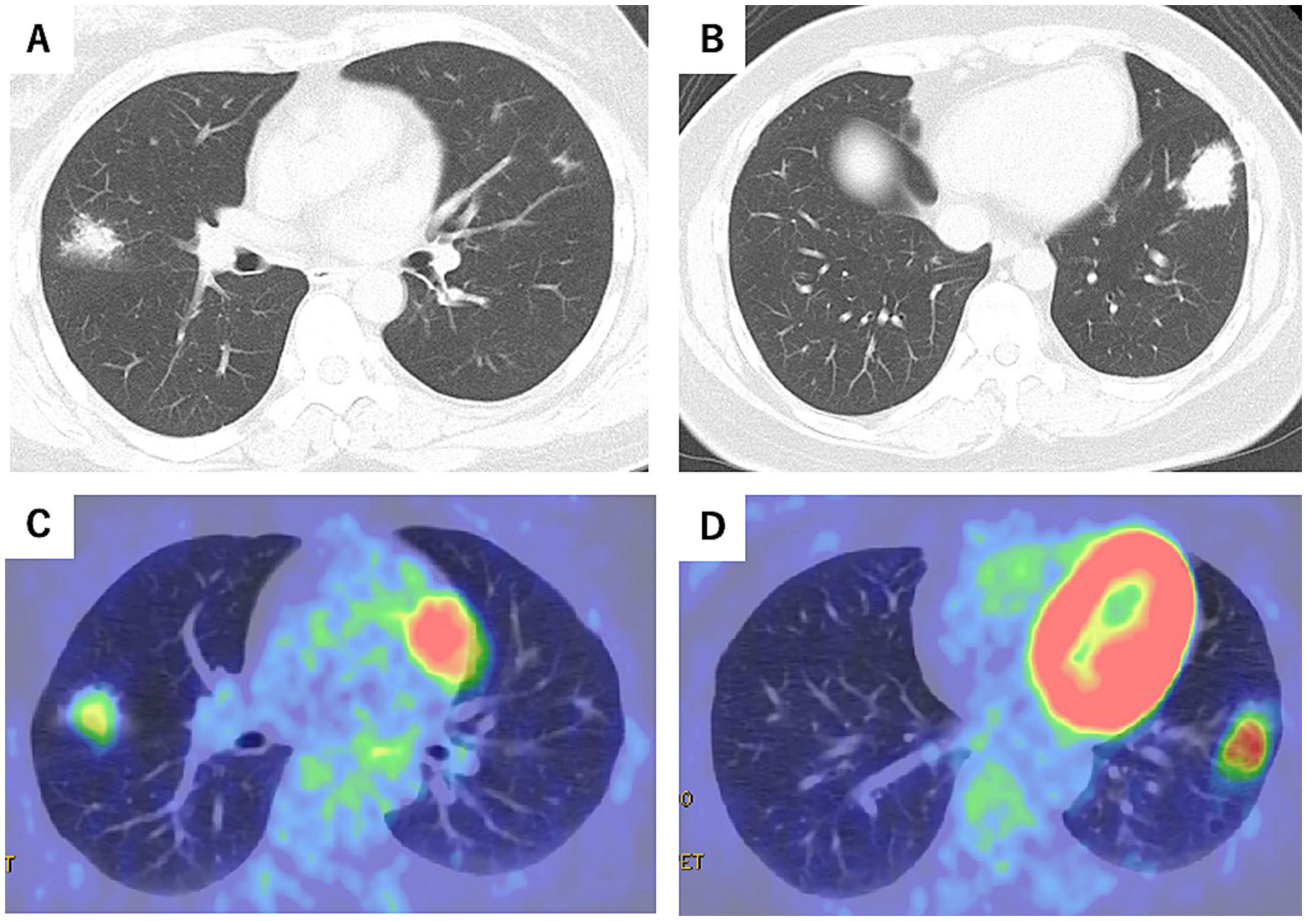
Chest CT at the initial visit (Day 0) **(A,B)** and positron emission tomography-computed tomography (PET-CT) scans **(C,D)** (October X-year). Irregular nodular lesions were observed in the right middle lobe (S4), measuring 20 mm, with a maximum standardized uptake value (SUVmax) of 2.78 on PET-CT. Similar nodular lesions in the left lower lobe (S8), measuring 35 mm, showed an SUVmax of 4.53.

At the first visit, chest CT revealed irregular nodular lesions in the right middle lobe (S4), measuring 20 mm, and in the left lower lobe (S8), measuring 35 mm. Positron-emission tomography/computed tomography (PET-CT) showed uptake with maximum standardized uptake value (SUVmax) 2.78 in the right middle lobe (S4) and SUVmax 4.53 in the left lower lobe (S8) (Figures 2A–D). Blood tests showed a mild elevation in CRP at 0.17 mg/dL, but tumor markers, including CEA, CYFRA, and Pro-GRP, were within normal limits. sIL-2R levels were also normal (381 U/mL). Sputum examination was negative for significant bacteria, including acid-fast bacilli, and fungi such as *Aspergillus*.

**Figure 3:**
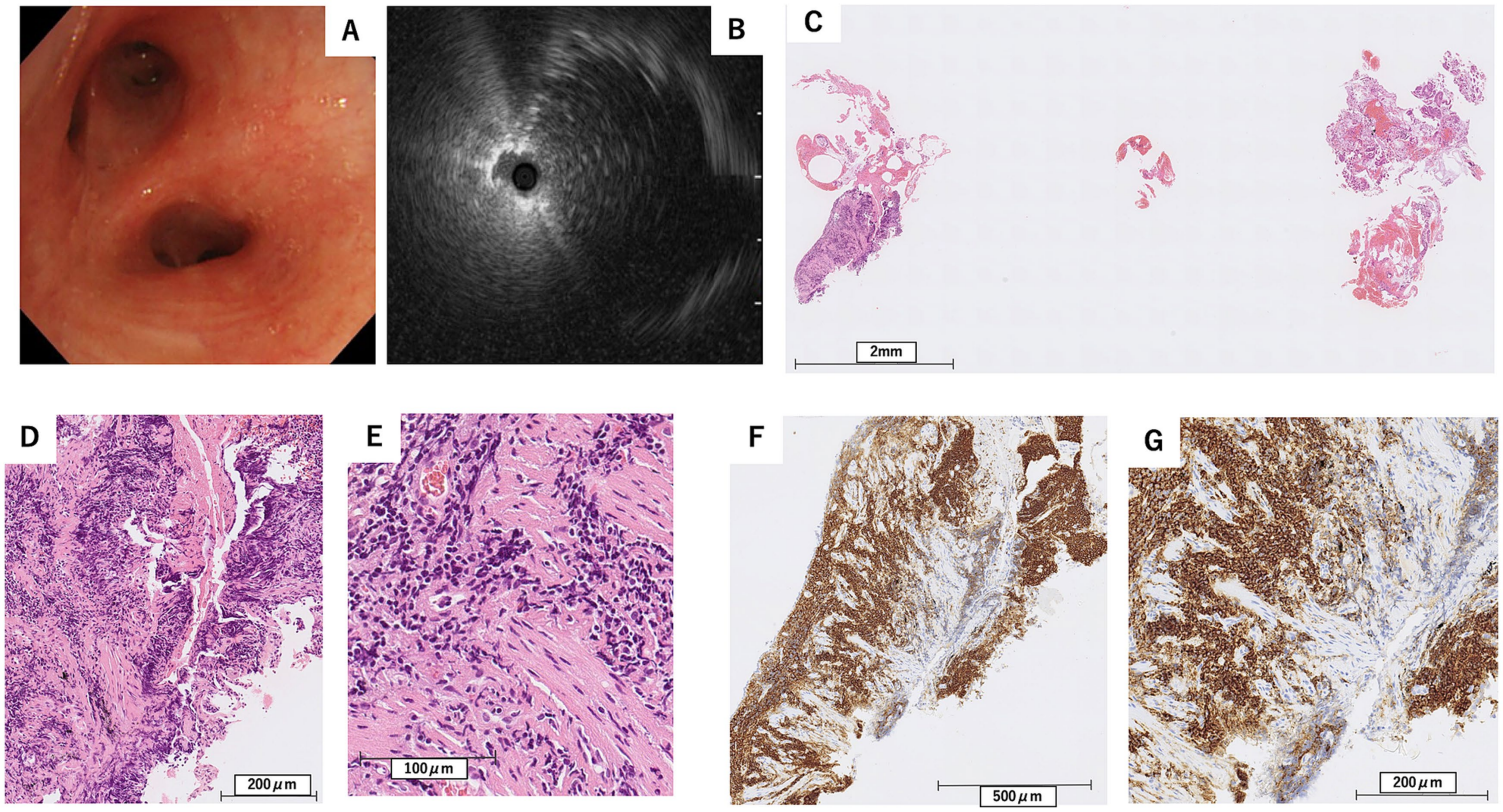
Findings from the first bronchoscopy **(A)**, and endobronchial ultrasound at the left B8b bronchus **(B)**, along with pathological examination results from the transbronchial lung biopsy **(C–G)**. **(C)** Hematoxylin–Eosin stain, scale bar = 2 mm, **(D)** Hematoxylin–Eosin stain, scale bar = 200 μm, **(E)** Hematoxylin–Eosin stain, scale bar = 100 μm, **(F)** Immunohistochemical stain for CD20, scale bar = 500 μm, **(G)** Immunohistochemical stain for CD20, scale bar = 200 μm. Lymphocytic infiltration positive for CD20 was observed in the interstitium **(F,G)**, while only a few cells were differentiated into plasma cells **(D,E)**.

To achieve a definitive diagnosis, flexible bronchoscopy under moderate sedation was performed in October X year (1 month after the initial visit) to obtain samples from the left lower and right middle lobes. In the left lower lobe, endobronchial ultrasound (EBUS) confirmed that the lesion was adjacent to the target lesion in the B8b branch (Figures 3A,B). Subsequently, a guide sheath transbronchial lung biopsy (GS-TBLB) was performed 10 times at this site using BF-1TQ290 (Olympus, Tokyo, Japan) and FB-231D (Olympus, Tokyo, Japan). The following day, a similar procedure was performed on the B4a branch of the right middle lobe, where EBUS confirmed the target lesion, and GS-TBLB was performed nine times at this site. Histopathological analysis of samples from both lobes showed interstitial lymphocytic infiltration, although many lymphocytes were degenerated. Immunohistochemical staining revealed a predominance of CD20-positive cells; however, few cells differentiated into plasma cells, and light chain restriction was unclear, suggesting no obvious evidence of lymphoma (Figures 3C–G).

Despite these findings, a definitive diagnosis was not reached based on imaging findings and lymphocytic infiltration observed on the pathological examination. Cryptogenic organizing pneumonia was considered, and treatment with prednisolone 30 mg/day was initiated in October X. Prednisolone treatment continued for 3 months, but the tumorous lesions persisted on subsequent chest CT scans (Figure 1). As a result, bronchoscopy was repeated for diagnostic evaluation.

**Figure 4:**
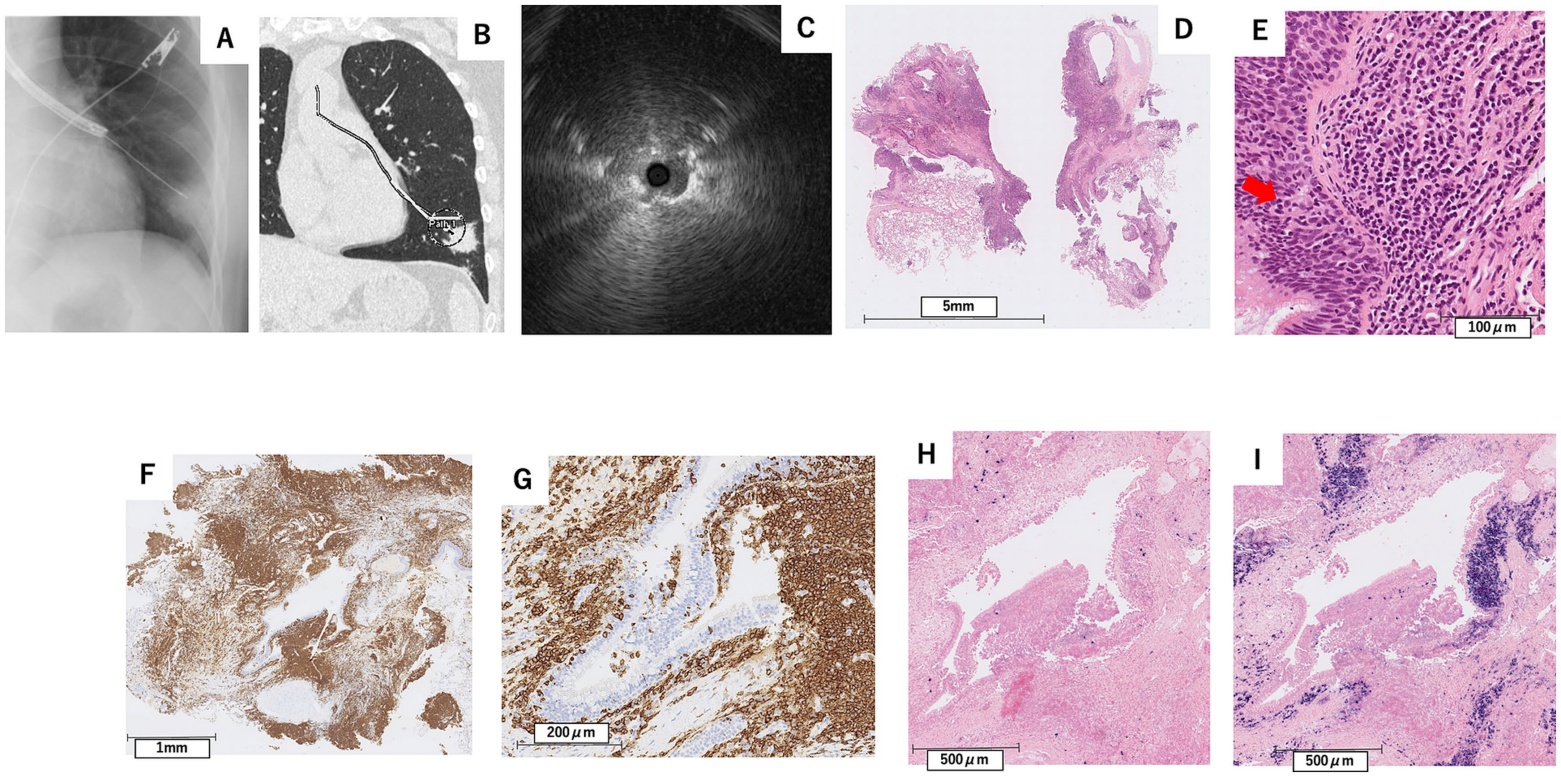
Findings from X-ray fluoroscopy during bronchoscopy in April X + 1 year **(A)**, CT image showing the planned biopsy route for transbronchial lung cryobiopsy **(B)**, and endobronchial ultrasound at the left B8b bronchus **(C)**, along with pathological examination results from the TBLC **(D–I)**. **(D)** Hematoxylin–Eosin stain, scale bar = 5 mm, **(E)** Hematoxylin–eosin stain, scale bar = 100 μm, **(F)** Immunohistochemical stain for CD20, scale bar = 1 mm, In-situ hybridization for kappa **(G)** and lambda **(H)** light chain mRNA, scale bar = 500 μm. Immunohistochemical staining showed positivity for CD20 in infiltrating lymphocytes **(F,G)**, observed infiltrating the bronchial epithelium in a lymphoepithelial lesion-like pattern **(E)**. In-situ hybridization showed *λ* dominant light chain restriction **(H,I)**.

In April X + 1 year (7 months after the initial visit), flexible bronchoscopy under moderate sedation was performed, and EBUS confirmed the adjacent lesion in the B8b branch (Figures 4A–C). Transbronchial lung cryobiopsy with a 2.0 mm probe (Erbe Elektromedizin, GmbH, Tuebingen, Germany) was performed twice. No complications, such as bleeding or pneumothorax, were observed after the procedure.

Pathological examination revealed diffuse infiltration of small lymphoid cells into the lung parenchyma and subepithelial bronchial tissue, with some cells differentiating into plasma cells. Immunohistochemical staining showed positivity for CD20 and bcl-2 in infiltrating lymphocytes, some of which infiltrated the bronchial epithelium, showing a lymphoepithelial lesion (LEL)-like pattern (Figures 4D–G). In-situ hybridization showed *λ* dominant light chain restriction (Figures 4H,I). Based on these findings, the patient was diagnosed with MALT lymphoma.

A subsequent bone marrow examination confirmed the absence of tumor infiltration. Because the patient was asymptomatic, showed no signs of organ dysfunction, and had no evidence of bone marrow involvement, a watch-and-wait strategy was adopted. The last CT follow-up was performed 21 months after the first visit and 14 months after the diagnosis. Although there has been a slight tendency toward enlargement, no symptoms have appeared. In consultation with a hematologist, we are continuing follow-up imaging tests every 3 months and planning to initiate rituximab treatment in the event of further lesion enlargement or symptom onset (Figure 1).

### Diagnostic assessment

At the time of the initial visit to our hospital, chest CT revealed irregular nodular lesions in the right middle lobe (S4), measuring 20 mm, and in the left lower lobe (S8), measuring 35 mm. PET-CT showed uptake with an SUVmax 2.78 in the right middle lobe (S4) and SUVmax 4.53 in the left lower lobe (S8). Based on the first bronchoscopy specimen, the differential diagnosis included lymphoproliferative disorders such as MALT lymphoma, as well as organizing pneumonia caused by reactive B-cell infiltration associated with infection or chronic inflammation.

Based on the specimen obtained from the second bronchoscopy (TBLC), in-situ hybridization demonstrated *λ*-dominant light chain restriction, leading to a definitive diagnosis of MALT lymphoma. The Ki-67 labeling index was 15%. Moreover, no morphological or clinical features suggestive of high-grade transformation were observed. According to the Lugano classification, this case was staged as stage IV due to the presence of two non-contiguous pulmonary lesions.

## Discussion

In this case report, during the first TBLB, the sample size was small, and while CD20-positive cells were observed, few cells showed differentiation into plasma cells, and light chain restriction was unclear. In contrast, the second TBLC yielded larger samples, which allowed for the identification of infiltrating lymphocytes, some of which infiltrated the bronchial epithelium showing lymphoepithelial lesions and light chain restriction, contributing to the diagnosis of pulmonary MALT lymphoma.

Primary pulmonary malignant lymphoma is a relatively rare disease, accounting for 3–4% of extranodal lymphomas and 0.5–1% of primary malignant lung tumors, with the majority being MALT lymphoma (6). Additionally, 30–50% of pulmonary MALT lymphoma cases are asymptomatic at diagnosis, and when symptoms are present, they are often nonspecific, such as cough or chest pain (12).

On radiological examination, pulmonary MALT lymphomas typically manifest as multiple or solitary lesions, including consolidation with air bronchograms, nodules, masses, ground-glass opacities (GGO), and diffuse interstitial lung disease patterns (13). Based on these radiological findings, differential diagnoses such as lung cancer and pulmonary infections, including tuberculosis, are considered. Therefore, while a definitive diagnosis through tissue biopsy is crucial, traditional bronchoscopy and CT-guided biopsies have a low diagnostic yield (7). Surgical lung biopsy using video-assisted thoracoscopy is considered the gold standard for obtaining adequate tissues. In this case, the initial CT findings suggested differential diagnoses, including lung cancer and other thoracic malignancies, as well as fungal and mycobacterial infections. Sputum and tumor marker examinations returned negative results. Consequently, TBLB was performed for pathological diagnosis, which revealed lymphocyte infiltration without any evidence of tumor cells or infectious agents, leading to the initial consideration of organizing pneumonia and subsequent steroid treatment.

However, the optimal treatment strategy for pulmonary MALT lymphoma remains unclear. Surgery or radiation therapy is preferred for localized cases, whereas chemotherapy or rituximab is viable for unresectable cases. In asymptomatic patients, a “watch and wait” approach may also be considered (14). Thoracoscopic resection is often performed in patients with solitary lesions for both diagnostic and therapeutic purposes. However, pulmonary MALT lymphoma is associated with a favorable prognosis, with a 5-year survival rate exceeding 90% and a potential for survival beyond 10 years (15). Consequently, approximately 70% of patients, particularly those asymptomatic at diagnosis, may not require immediate treatment and can be managed with observation alone without disease progression (16). Given these situations, for asymptomatic patients, considering the invasiveness of surgery, it is preferable to achieve a diagnosis using less invasive methods such as bronchoscopy when possible. In this case, a definitive diagnosis of MALT lymphoma was achieved through TBLC. Since the patient remained asymptomatic, and no enlargement of the lesions was observed during follow-up, we continued with observational management.

In a previous report on pulmonary MALT lymphoma using a 1.1 mm cryobiopsy probe (8), histopathological diagnosis could not be obtained in two out of four cases. In contrast, in our case, a 2.0 mm probe was used, suggesting that probe size may be an important factor for achieving histopathological diagnosis. Although a recent case of tumor seeding following cryobiopsy has been reported (17), there was no evidence of tumor seeding in our case, such as rapid tumor growth after the procedure. However, further long-term evaluation is warranted to assess the safety of this technique.

Compared with conventional TBLB, TBLC carries a higher risk of bleeding and pneumothorax. A meta-analysis targeting interstitial lung disease reported major bleeding rates of 9.9% and pneumothorax treated with a chest tube in 5.6% (18). However, a study assessing the efficacy and safety of TBLC in 13 patients with lymphoproliferative disease found no instances of major bleeding or pneumothorax (8). While biopsy locations and the number of biopsies may differ between interstitial lung disease and other conditions, in this case, TBLC did not result in major bleeding or pneumothorax, suggesting that TBLC is relatively safe and effective for diagnosing MALT lymphoma. In the aforementioned study of 13 cases, all patients were deeply sedated with propofol and intubated with a rigid tracheoscope. In contrast, we conducted examinations using a flexible bronchoscope under moderate sedation, which is considered less invasive.

The strength of our report lies in being the first case in Japan to demonstrate diagnosis by transbronchial lung cryobiopsy after unsuccessful transbronchial lung biopsy in the same patient. Additionally, our case highlights the safety of peripheral cryobiopsy in pulmonary MALT lymphoma. Limitations include it being a single case with limited follow-up. The reason for diagnostic failure in the first biopsy may be that, despite performing nine GS-TBLB procedures, the specimens were insufficient in size, and only a few cells showed differentiation into plasma cells. Additionally, light chain restriction could not be confirmed. In contrast, the subsequent cryobiopsy yielded sufficiently large samples that demonstrated infiltrating lymphocytes, some of which invaded the bronchial epithelium forming lymphoepithelial lesions and exhibited light chain restriction, leading to a definitive diagnosis.

## Conclusion

In this report, we present a case where pulmonary MALT lymphoma was diagnosed using TBLC after TBLB failed to provide a definitive diagnosis. Pulmonary MALT lymphoma is associated with a favorable prognosis. Given the option of observation without post-diagnostic treatment, minimally invasive diagnostic approaches are preferable to surgery. When considering pulmonary MALT lymphoma as a differential diagnosis, cryobiopsy is useful for obtaining a definitive diagnosis.

## Data Availability

The raw data supporting the conclusions of this article will be made available by the authors, without undue reservation.
